# The Associations Between the Swimming Speed, Anthropometrics, Kinematics, and Kinetics in the Butterfly Stroke

**DOI:** 10.3390/bioengineering12080797

**Published:** 2025-07-25

**Authors:** Mafalda P. Pinto, Henrique P. Neiva, Tatiana Sampaio, João P. Oliveira, Daniel A. Marinho, Tiago M. Barbosa, Jorge E. Morais

**Affiliations:** 1 Department of Sport Sciences, University of Beira Interior, 6201-001 Covilhã, Portugal; mafalda.maria.pinto@ubi.pt (M.P.P.); henriquepn@gmail.com (H.P.N.); tatiana_sampaio30@hotmail.com (T.S.); jpco-2001@live.com.pt (J.P.O.); marinho.d@gmail.com (D.A.M.); 2 Research Centre in Sports, Health and Human Development (CIDESD), 6201-001 Covilhã, Portugal; 3 Department of Sport Sciences, Instituto Politécnico de Bragança, 5300-253 Bragança, Portugal; barbosa@ipb.pt; 4 Research Centre for Active Living and Wellbeing (LiveWell), Instituto Politécnico de Bragança, 5300-253 Bragança, Portugal

**Keywords:** swimming, performance, butterfly, stroke mechanics, thrust

## Abstract

There is scarce information about what characterizes the swimming speed in the butterfly stroke and the role of thrust in its characterization and prediction. The aim of this study was to compare the fastest and poorest butterfly swimmers based on a set of anthropometric, kinematic, and kinetic variables and to identify the swimming speed predictors. Eight young male swimmers were divided into two equal groups (each group comprising four swimmers). The swimming speed, as well as a set of anthropometric, kinematic, and kinetic variables, were measured. The swimming speed presented significant differences between the groups (*p* = 0.011, *d* = 2.18). The stroke frequency (kinematics, *p* = 0.027, *d* = 1.69) and thrust (kinetics, *p* = 0.034, *d* = 1.57) also presented significant differences between the groups. The swimming speed presented significant correlations with the stroke index (*r_s_* = 0.83, *p* = 0.011) and thrust (*r_s_* = 0.83, *p* = 0.011). The swimming speed was predicted by a combination of the stroke frequency and thrust (R^2^ = 0.84, *p* = 0.010). Coaches and athletes must be aware that combining fast stroke frequencies and the generation of greater thrust leads to the fastest swimming speeds.

## 1. Introduction

Understanding how the swimming speed can be improved and which are its main determinants is a major aim among swimming researchers and coaches. Being a time-based sport, speed is the most critical performance indicator in competitive swimming, influencing the outcomes across various race distances and swim strokes [1,2]. Being a measure of performance, coaches, researchers, and practitioners put a lot of focus on enhancing the swimming speed through training and technical refinement.

The swimming speed is influenced by anthropometrical, physiological, biomechanical, motor control, and other factors [3]. Overall, the literature reports that swimmers with bigger body dimensions (anthropometrics) [2,4], a better swimming technique—i.e., the fastest stroke frequencies, a larger stroke length, smaller speed fluctuations, and a greater stroke index [5,6]—and greater energetics or efficiency [7,8] are more likely to deliver better performances. Within the biomechanical scientific field, kinetics mostly involves the study of hydrodynamics [9,10]. Here, two forces can be studied and analyzed: drag and thrust. The drag is considered the resistance to a swimmer moving through water [11]. This can be considered passive (water resistance produced during the displacement of a towed body) or active (water resistance produced while swimming) [12]. The thrust is related to the propulsive forces, i.e., the resultant force (net effect of the horizontal, vertical, and lateral components) generated by the swimmer through the actions of the upper and lower limbs to promote forward motion [11]. The drag has been widely studied in swimming, whether using experimental [13,14], numerical [15,16], or analytical methods [17,18]. Research in swimming has claimed that the absolute value of the drag may not be the best way to understand a swimmer’s hydrodynamic profile [19]. On the other hand, the drag coefficient seems to better represent swimmers’ overall hydrodynamic profiles since it is less dependent on the swimming speed [19].

As for the thrust, its measurement and interpretation play an important role in monitoring training and guiding performance assessments [20,21]. However, the aquatic environment’s inherent complexity poses significant challenges for accurate force measurement. Consequently, the thrust has mostly been measured using indirect experimental methods, for example, tethered methods [22], or through estimations based on kinematic data [23]. Nonetheless, advancements in technology have improved both the measurement techniques and methodologies applied in this field, i.e., aquatic environments [24,25]. Nowadays, swimming researchers mostly use pressure sensors to measure the hand force generated by swimmers during their swim stroke [10,26]. These can be used with [27] or without cabling [10]. More importance is being given to the upper limbs’ thrust, in comparison to that of the lower limbs, because it has been shown that the former are more responsible for the swimming speed than the latter [28]. Wearables are also being used in the aquatic environment [24]. These are electronic devices that are designed to be worn on the body, either as accessories or as part of clothing. They typically include sensors and wireless communication features, allowing them to collect data, track activities, and interact with other devices or systems [29]. In the case of swimming research, these wearables are mainly inertial measurement units (IMUs). Water-based activities necessitate sensors to be hermetically sealed to ensure they are water-resistant or waterproof. Advancements in technology have led to the miniaturization of sensors, which is particularly beneficial in aquatic environments as it helps minimize the drag. Additionally, for precise kinematic and kinetic data collection, sensors can be positioned on body segments that do not contribute to increased drag, interfere with natural movement, or restrict the participant’s range of motion [30]. Therefore, based on all these advantages, wearables are now being widely used in a swimming research context [31,32,33].

In swimming research, the front-crawl stroke is the most studied for several reasons (e.g., it is the fastest, the most efficient, less complex, and the one in which swimmers competitively participate since early childhood). On the other hand, less information exists about the butterfly stroke. This is more complex in terms of motor control than front-crawl [34], and it is the most demanding stroke energetically [35]. Consequently, the body of knowledge about butterfly performance, at least compared with that for front-crawl, is limited. Nonetheless, it has been shown that the best butterfly swimmers, as well as the best swimmers using the remaining strokes, are characterized by having higher anthropometrics and better kinematic patterns [36,37]. Regarding the thrust in the butterfly stroke, as in swimming in general, this is usually measured based on tethered methods [22,38]. In regard to thrust measurement using wearables and direct methods, the literature has scarce information about this topic in swimming in general (most studies are related to the front-crawl stroke) [26,39], and even less can be found on the butterfly stroke [40]. Nonetheless, it has been noted that the fastest swimming speeds are related to greater thrust [40,41]. Thus, one can argue that understanding how the thrust can determine the swimming speed can be useful for coaches and practitioners.

Overall, by identifying and enhancing the variables that influence or have a greater impact on the swimming speed in the butterfly stroke, the overall performance can be improved, leading to meaningful gains in a competitive context. Therefore, the aim of this study was to compare the fastest and poorest butterfly swimmers based on a set of anthropometric, kinematic, and kinetic variables and to identify the swimming speed predictors. It was hypothesized that the thrust would be identified as a swimming speed predictor.

## 2. Materials and Methods

### 2.1. Participants

The study sample comprised eight male swimmers, evenly divided into two groups (poorest performers: N = 4, age = 17.4 ± 0.7 years, World Aquatic Points for the 50 m butterfly event in a long-course swimming pool = 553.6 ± 17.6 points; best performers: N = 4, age = 18.2 ± 1.3 years, World Aquatic Points for the 50 m butterfly event in a long-course swimming pool = 656.0 ± 32.6 points). They were selected from a national team that regularly competed at national and international events. The sample included age-group national record holders, national champions, and swimmers who were part of a national talent identification program (Tier 3 athletes) [42]. They followed a training regimen of six to nine sessions per week. Data collection took place during their peak performance phase after their second macro-cycle.

To be eligible, the swimmers had to be specialists in the butterfly stroke and be free from any physical limitations, such as injuries sustained in the past six months, that could impact their performance. The participants received both verbal and written explanations of the study before providing written informed consent. All the procedures adhered to the ethical guidelines outlined in the Declaration of Helsinki for research involving human subjects and were approved by the Ethics Committee of the Instituto Politécnico de Bragança (No. P547664-R678573-D2082555).

### 2.2. Study Design

**Figure 1:**
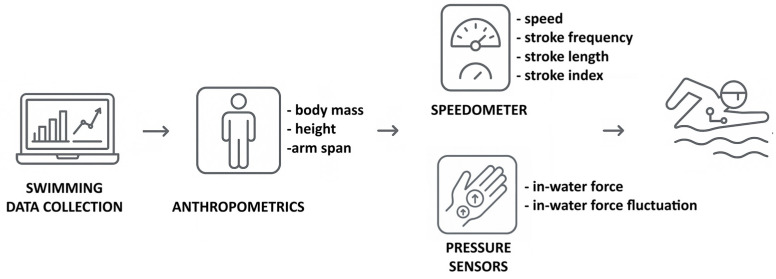
Visual representation of the data collection setup.

An expert evaluator (with several years of experience running the procedures, which were based on those of the International Society for the Advancement of Kinanthropometry) measured the swimmers’ anthropometrics in a gym [43]. These variables included their body mass, height, and arm span. Before in-water data collection, the swimmers completed a standardized warm-up specifically designed for sprint swimming, as outlined by others [44]. Following an auditory cue, each swimmer performed three maximum-effort 25 m butterfly trials, starting with a push-off. A 30 min recovery period was taken between each trial to ensure full recovery. The fastest trial, based on the swimming speed, was selected for analysis. Only three consecutive stroke cycles beyond the 10 m mark were examined to eliminate any potential advantages from the wall push-off. Additionally, the swimmers were instructed to avoid breathing during these stroke cycles to maintain consistent stroke coordination and technique, preventing any negative impacts on their swimming speed [45]. The swimming speed (v), stroke frequency (SF), stroke length (SL), stroke index (SI), thrust (F), and thrust fluctuation (dF) were measured for further analysis. Figure 1 depicts the data collection setup.

### 2.3. Anthropometrics

The swimmers’ body mass was measured with the swimmers in an upright position with a digital scale (Tanita, MC 780-P MA, Tokyo, Japan) to the nearest decimal place. Their height was also measured vertically from the vertex to the floor with a digital stadiometer (SECA, 242, Hamburg, Germany) to the nearest decimal place. The swimmers were placed in an orthostatic position for the arm span assessment. Both their arms were in lateral abduction at a 90° angle to their trunk, and their arms and fingers were fully extended. The distance between the tip of each third finger was measured with a flexible anthropometric tape (RossCraft, Surrey, Canada). A test/retest evaluation was performed using the Intraclass Correlation Coefficient (ICC). This was very high (ICC = 0.99).

### 2.4. Kinematics

A mechanical device (SpeedRT speedometer, ApLab, Rome, Italy) was used to assess the swimming speed, with its string attached to the swimmers’ waists [46]. This speedometer recorded the displacement and speed at a frequency of 100 Hz. The collected speed–time data were then transferred to signal processing software (AcqKnowledge v. 3.9.0; Biopac Systems, Santa Barbara, Goleta, CA, USA). Following residual analysis [47], the signal was processed using a 4th-order Butterworth low-pass filter with a 5 Hz cut-off frequency. Additionally, a video camera (GoPro Hero Black 7, GoPro, San Mateo, CA, USA) was positioned at a fixed location in the middle section of the swimming pool [48]. The camera was synchronized with the mechanical device to capture footage in the sagittal plane and identify the entry and exit points of the hand. The mechanical device was synchronized with the video camera using a bright light-emitting diode flash embedded in the device. At the moment that the mechanical device was started, the light turned on, and it was in the video camera’s field of view. The flash was recorded in the video, creating a visible timestamp. During post-processing, the frame showing the flash was used to align the video with the mechanical data. This method was simple, accurate, and non-invasive [49]. The software was used to obtain the swimming speed (in m/s) during three consecutive stroke cycles (based on the hand’s entry and exit). The SF was measured by calculating the number of cycles per unit of time from the time required to complete a full cycle (f = 1/t), then converted to Hz. The SL (in m) was calculated as SL = v/SF. The SI (in m^2^/s) was calculated as SI = v ∙ SL [50].

### 2.5. Kinetics

The thrust (in N) was measured using a set of wearable paddles (PoolShark, Tampere, Finland). This sensor system captures various kinetic parameters during swimming strokes [51]. This system comprises three key components: the wearables, the PoolShark Session Manager mobile application for data recording, and the Analysis Center (URL: https://sharksensors.com/; accessed on 5–9 May 2024) for data storage and analysis. The wearable units are secured to the swimmers’ hands using silicone straps. They measure the applied in-water force using two pressure sensors and track movement using a 9-axis IMU, with a sampling rate of 100 Hz. The PoolShark Session Manager is the interface between the wearables and a mobile device, facilitating data recording and uploading to the Analysis Center. Once uploaded, the Analysis Center processes the recordings, visualizes the performance metrics, and provides immediate feedback for training. Additionally, it offers the option to download the data for further analysis. The algorithm used for the calculations has not been publicly disclosed. The processed data is generated using a closed Matlab GUI (Graphical User Interface) developed by the company (second version). As a result, access to the raw data is restricted, and users cannot modify the calculation constants within the algorithm. Notwithstanding, this system presented non-significant differences to and very high agreement [52] with other apparatus that measure the in-water force and are commonly described in the literature [27,53]. The average value of the thrust produced by both upper limbs was used for analysis. Afterwards, the dF (in %) was calculated as dF = standard deviation/mean ∙ 100 [41].

### 2.6. Statistics

The Shapiro–Wilk test was used to assess the sample distribution. All the variables showed a normal distribution. The mean plus standard deviation was calculated as descriptive statistics. An independent sample *t*-test was used to compare the groups (poorest vs. best performers). The mean differences with 95% confidence intervals (95% CIs) were computed. The alpha level was set at *α* = 0.05. Cohen’s d was used to estimate the effect size to be (i) trivial if 0 ≤ *d* < 0.20; (ii) small if 0.20 ≤ *d* < 0.60; (iii) moderate if 0.60 ≤ *d* < 1.20; (iv) large if 1.20 ≤ *d* < 2.00; (v) very large if 2.00 ≤ *d* < 4.00; and (vi) nearly decisive if *d* ≥ 4.00 [54]. The Spearman correlation (*r_s_*) coefficient (*p* < 0.05) was applied to examine the relationship between the swimming speed and the remaining variables, including both the strength and direction of these correlations. Simple linear regression (backward method) was used to test the swimming speed predictors [55]. The coefficient of determination (R^2^) was used to understand the magnitude of the relationship. Qualitatively, this was defined to be very weak if R^2^ < 0.04, weak if 0.04 ≤ R^2^ < 0.16, moderate if 0.16 ≤ R^2^ < 0.49, high if 0.49 ≤ R^2^ < 0.81, and very high if 0.81 ≤ R^2^ <1.0. The standard error of estimation (SEE) was also considered.

## 3. Results

The descriptive data for the two groups are presented in Table 1. The swimming speed presented a significant difference between the groups with a very large effect size (mean difference = −0.15 m/s, 95CI = −0.26 to −0.03, *t*-test = −3.086, *p* = 0.011, *d* = 2.18). None of the anthropometric variables presented significant differences between the groups. The swimmers included in the fastest group did present meaningful differences in their anthropometric lengths (i.e., height and arm span). Besides the swimming speed, the SF (kinematics) and thrust (kinetics) also presented significant differences between the groups. Of these, the SF was the one showing the greatest difference, with a large effect size (mean difference = −0.09 Hz, 95CI = −0.18 to 0.002, *t*-test = −2.391, *p* = 0.027, *d* = 1.69).

**Figure 2:**
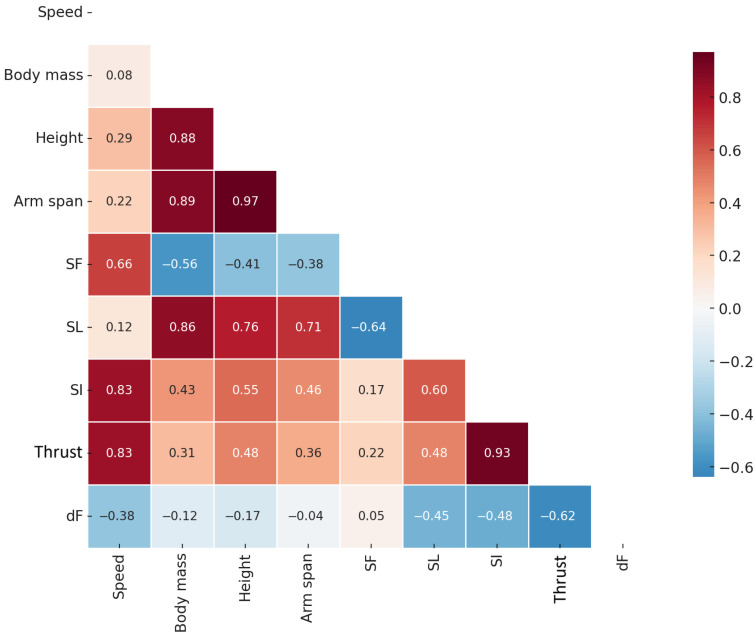
Heatmap of Spearman’s correlations. The color grading, from blue to red, indicates stronger and more significant correlations. SF—stroke frequency; SL—stroke length; SI—stroke index; dF—thrust fluctuation.

Figure 2 presents a heatmap of Spearman’s correlations (*r_s_*) between all the variables measured. The swimming speed presented significant correlations with the SI (*r_s_* = 0.83, *p* = 0.011) and thrust (*r_s_* = 0.83, *p* = 0.011).

**Figure 3:**
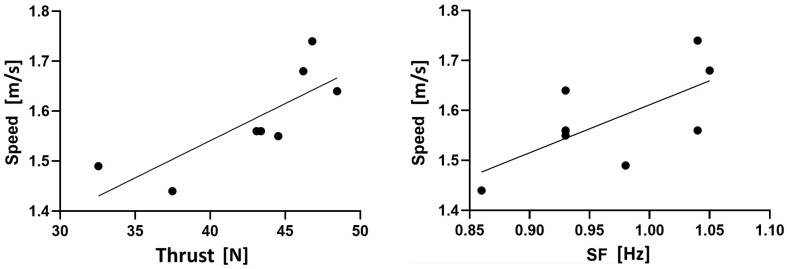
Relationships between swimming speed and thrust and between swimming speed and stroke frequency (SF).

The simple linear regression retained the thrust (β = 0.012; *p* = 0.016) and SF (β = 0.689; *p* = 0.049) (R^2^ = 0.84, *p* = 0.010; SEE = 0.04) as significant predictors of the swimming speed. The individual relationships are presented in Figure 3. The thrust presented a strong relationship with the swimming speed (R^2^ = 0.63), while the SF presented a moderate relationship with it (R^2^ = 0.45). The final prediction equation was

(1) $$Speed=0.389+\left(0.012\cdot Thrust\right)+\left(0.689\cdot SF\right)$$

where *Speed* is the swimming speed (in m/s), *Thrust* is the thrust generated (in N), and *SF* is the stroke frequency (in Hz).

## 4. Discussion

Thrust measurement is crucial in swimming because it directly reflects a swimmer’s ability to propel themselves efficiently through water, ultimately influencing their speed and performance. However, accurately measuring the thrust in real time has traditionally been challenging due to the dynamic and aquatic environment. Wearable technology offers a promising solution by enabling continuous in-water data collection. The measurement of the thrust and its relationship with other swimming determinants will help to increase the body of knowledge about swimming performance. The main findings of the present study are that the fastest swimmers in the butterfly stroke presented a significantly higher SF, larger SI, and greater thrust. The combination of the SF and thrust also predicted the swimming speed. This corroborates our hypothesis, as the thrust was identified as a swimming speed predictor.

Regarding the anthropometric comparison, non-significant differences were noted between the groups. Overall, the literature reliably reports that the fastest swimmers are bigger and taller and have longer levers than their slowest counterparts [4,56]. In a study that evaluated young adult swimmers in the four swim strokes (backstroke, breaststroke, butterfly, and front-crawl) and medley (i.e., a race where a swimmer or team of swimmers uses all four strokes), it was noted that there are body features that are common within swimmers who compete in all these events (such as their upper body circumference and waist circumference) [2]. This enhances the “v-shape” morphology of the best swimmers, regardless of the event or swim stroke. On the other hand, the arm length varied significantly between swimmers who participated in all the swim strokes and the medley. It was noted that butterflyers (as well as freestylers and backstrokers) with longer arm lengths were more likely to present the fastest swimming speeds [2]. Others noted that in young adult swimmers, the fastest butterflyers were more likely to have a greater body mass and lean body mass [36]. Once again, despite non-significant differences being observed in the present study, the fastest group was taller and had a longer arm span than the slowest group.

Of all the kinematic variables measured, the stroke frequency (SF) was the only one that presented significant differences between the groups, where the fastest group presented the highest SFs. Indeed, the literature reports that for maximal trials (i.e., all-out speeds) and other race paces, the SF is a variable that always presents differences between groups (fastest SFs in the fastest group) [37]. It has also been shown that the SF presents a significant and positive correlation with the swimming speed in the butterfly swim stroke [36]. Thus, one can argue that the SF seems to be the variable that best determines the swimming speed (in all-out trials) from a kinematic perspective. However, less is known about how the thrust (kinetics) distinguishes the best from the poorest performers in swimming, particularly in butterfly. As mentioned, taking a general perspective, great thrust leads to fast swimming speeds [20]. Few studies can be found about the thrust in the butterfly stroke specifically [27,40]. For instance, Kudo and co-workers aimed to investigate if the hand force (thrust) when performing the front-crawl stroke was different from that for the butterfly [40]. The authors noted that there were similarities and differences in the hand force between front-crawl and the butterfly stroke [40]. In another study aiming to understand the inter-limb differences in the thrust, it was shown that young adult butterfliers presented significant differences between their dominant and non-dominant sides [27]. As far as our understanding goes, only one study has compared the performance (swimming speed) and thrust [41]. The authors simultaneously compared the swimming speed and thrust between boys and girls (which led to a two-level interpretation, as the boys were faster than the girls). As well as significant differences in the speed, a significant difference was also noted in the thrust. This showed that the fastest swimmers also delivered the greatest thrust [41]. Conversely to the findings of Pereira and co-workers [27], this latter study [41] did not find significant differences between the upper limbs. Based on these results, it seems that the literature lacks comparisons of the best and poorest performers regarding the thrust.

The same lack of information in the literature can be seen for the relationship between the thrust and speed and how the thrust can determine the speed. The present data indicate that the thrust presented a positive and significant correlation with the swimming speed. This means that swimmers who generated greater thrust were more likely to deliver the fastest swimming speeds. As mentioned earlier, there are no studies (at least based on our knowledge) that have conducted this type of analysis using wearables and in the butterfly swim stroke. On the other hand, information obtained using equipment without [10] and with cabling [57] has been reported about this relationship for the front-crawl stroke, as well as information obtained using tethered methods [22] for the butterfly stroke. The studies by Pereira [27] and co-workers and Sampaio and co-workers [58] did use cabled equipment to measure swimmers’ thrust in the butterfly stroke, but in both cases, the aim was to compare the thrust between the upper limbs, i.e., to understand the inter-limb difference. Nonetheless, based on studies of the front-crawl stroke (which is similar to the butterfly stroke but unilateral [59]), it has been noted that the thrust presents a positive and significant relationship with the swimming speed [10]. This has also been noted in the butterfly stroke, but using tethered methods [22]. Thus, it seems that swimmers who generate more thrust are more likely to deliver the fastest swimming speeds. It must also be mentioned that, as well as the thrust, the stroke index (SI) also presented a positive and significant correlation with the speed (despite the non-significant differences between the best and poorest performers). This variable (SI) is an index that measures the amount of distance covered during each stroke cycle and is considered a swimming efficiency indicator [60]. Once again, there is solid information about this for the front-crawl stroke [61,62], but less information available for the butterfly stroke [61]. In a study by Morais and co-workers [61], the authors aimed to characterize the stroke kinematics of male swimmers competing in the four major 50 m events by comparing the fastest and the slowest swimmers. Regarding the SI, the authors noted that the fastest butterfly swimmers (with the fastest swimming speeds) presented the greatest SI in all the sections of the race in comparison to their slowest counterparts. This was also noted for the remaining strokes, i.e., backstroke, breaststroke, and front-crawl. Curiously, the thrust also presented a positive and significant correlation with the SI. If the SI is considered a variable that measures the amount of distance covered during each stroke cycle, one can argue that swimmers who generate more thrust are more prone to covering a longer distance per stroke.

The thrust and SF were identified as predictors of the swimming speed in the butterfly swim stroke, where both variables combined explained 84% of the speed’s variance. Once again, and as far as our understanding goes, there is no pre-existing information about how the thrust can determine the swimming speed in the butterfly stroke obtained using wearables. Information has been reported about the relationship between the thrust and speed in the butterfly stroke, where the thrust explained 82% of the speed’s variance [22]. However, the measurement of the thrust was based on tethered methods, which can lead to misleading results [63]. On the other hand, another study aimed to predict the main determinants of the swimming speed in the butterfly stroke based on a set of variables including the thrust and SF (among others) [41]. The authors noted that both the thrust and SF (along with the speed fluctuations and sex) emerged as predictors of the swimming speed. These findings are aligned with the present results, where the highest cadences (SFs) in combination with greater thrust led to the fastest speeds. This indicates that, at least in maximal trials, butterfliers are more likely to achieve the fastest speeds by generating greater thrust and, at the same time, achieving the highest cadences.

Overall, it was noted that the fastest swimmers in the butterfly stroke may not present significantly bigger body dimensions (despite having a larger body size) but tend to present a higher SF and greater thrust than their slowest counterparts. The thrust and SF also emerged as predictors of the swimming speed, indicating that this combination influences swimmers’ speed. Thus, at least for maximal trials or all-out events, coaches and athletes must be aware that increasing the SF and, at the same time, generating more thrust results in the fastest swimming speeds. Using wearables and measurement technologies in water activities, specifically in swimming, is of paramount importance to collect data related to swimming performance. In this case, wearable pressure sensors incorporating IMUs allow for the direct measurement of the thrust without compromising the swimmers’ stroke mechanics or technique. This study expands a bit more on the understanding of swimming performance by focusing on the direct relationship between the swimming speed and thrust. This research indicates that the thrust is a meaningful determinant of the swimming speed. Its novelty lies in quantifying the thrust in a way that correlates meaningfully with performance outcomes, offering a more targeted metric for performance analysis. Coaches can use this insight to tailor strength and technique drills that prioritize effective force application during the stroke cycle. Moreover, by incorporating thrust-focused feedback, coaches can help swimmers refine their technique in a data-driven way. Ultimately, this approach allows for more personalized training strategies aimed at maximizing the thrust, and therefore the speed, leading to more competitive performance outcomes. Using technology in sports measurements is now essential for providing accurate, real-time data that enhances performance analysis and optimizes training strategies. In modern athletics, small differences can determine the competitive outcomes, and technology offers a means to obtain the information needed to monitor and improve performance determinants.

The main limitation of this study can be considered the small sample size. A G*Power (v3.1) analysis [64] indicated that 15 participants were needed to detect a moderate effect size (r = 0.60) with 80% power (α = 0.05) for a “correlation: bivariate normal model” statistical test. Consequently, the associations from the present analysis must be considered with caution [65]. However, one must claim that it is challenging to find expert butterfly swimmers whose performance would not introduce potential data inconsistencies. The butterfly stroke is highly technical and physically demanding, making it difficult to isolate and control errors stemming from subtle technique variations or fatigue-related performance fluctuations. Future studies could (i) focus on increasing the body of knowledge about the relationship between the swimming speed and thrust in the butterfly stroke at several race paces and in female swimmers and (ii) seek to understand how the thrust affects the energetic profile of swimmers, also at several race paces, and compare the thrust of swimmers in breathing and non-breathing conditions. This comparison could provide more insights into the studied variables. Ultimately, it would be interesting to determine wearables’ performance during long-distance swimming.

## 5. Conclusions

The fastest butterfly swimmers were characterized by having better kinematics (higher SF) and greater thrust than their slowest counterparts. The SF, SI, and thrust were strongly correlated with the swimming speed, and the SF and thrust emerged as predictors of the swimming speed. Coaches and athletes now have more information about what distinguishes the fastest from the slowest male swimmers in the butterfly stroke and what the main determinants of the swimming speed are in maximal trials.

## Figures and Tables

**Table 1:** Descriptive statistics (mean ± standard deviation—SD) of all variables measured. It also presents information about comparison between groups (95CI—95% confidence interval).

		Mean ± SD Poorest Performers	Mean ± SD Best Performers	Mean Difference (95CI)	*t*-Test (*p*-Value)	Effect Size (Descriptor)
Anthropometrics	Body mass [kg]	77.6 ± 5.4	76.7 ± 13.9	0.88 (−17.43 to 19.18)	0.117 (0.455)	0.08 (trivial)
	Height [cm]	182.0 ± 8.3	186.7 ± 11.7	−4.75 (−22.29 to 12.79)	−0.663 (0.266)	0.47 (small)
	Arm span [cm]	186.0 ± 5.8	190.5 ± 9.6	−4.50 (−18.32 to 9.32)	−0.797 (0.228)	0.56 (small)
Kinematics	Speed [m/s]	1.51 ± 0.05	1.65 ± 0.07	−0.15 (−0.26 to −0.03)	−3.086 (0.011)	2.18 (very large)
	SF [Hz]	0.93 ± 0.05	1.02 ± 0.05	−0.09 (−0.18 to 0.002)	−2.391 (0.027)	1.69 (large)
	SL [m]	1.63 ± 0.07	1.63 ± 0.11	0.001 (−0.16 to 0.17)	0.009 (0.497)	0.01 (trivial)
	SI [m^2^/s]	2.47 ± 0.16	2.71 ± 0.26	−0.24 (−0.62 to 0.14)	−1.533 (0.088)	1.08 (moderate)
Kinetics	Thrust [N]	39.48 ± 5.56	46.14 ± 2.24	−6.65 (−13.99 to 0.69)	−2.218 (0.034)	1.57 (large)
	dF [%]	63.36 ± 8.37	57.17 ± 6.38	6.19 (−6.68 to 19.07)	1.177 (0.142)	0.83 (moderate)

SF—stroke frequency; SL—stroke length; SI—stroke index; dF—thrust fluctuation.

## Data Availability

The data presented in this study are available on request from the corresponding author. The data are not publicly available due to privacy or ethical restrictions.
